# Analyzing ageism in the district of La Matanza, Province of Buenos Aires Argentina: a design for a study and interventions to reverse inequality and discrimination towards old age

**DOI:** 10.1002/alz.084019

**Published:** 2025-01-09

**Authors:** Carolina Feldberg, Paula Daniela Hermida, Silvia Deborah Ofman, María Florencia Tartaglini, Irrazabal Natalia, Rosario Quian, Cecilia Serrano, Agustina Pollard

**Affiliations:** ^1^ Conicet‐ Ineba, Buenos Aires Argentina; ^2^ Ineba/Conicet, BUENOS AIRES Argentina; ^3^ CONICET, BUENOS AIRES Argentina; ^4^ Ineba ‐ Conicet, CABA Argentina; ^5^ Instituto de Neurociencias Buenos Aires (INEBA‐CONICET), Argentina, Ciudad Autónoma de Buenos Aires Argentina; ^6^ Universidad de Palermo, Buenos Aires Argentina; ^7^ Ineba, caba Argentina; ^8^ Cesar Milstein Hospital, Buenos Aires Argentina; ^9^ Universidad de Buenos Aires Facultad de Psicologia, caba, Buenos aires Argentina

## Abstract

**Background:**

The stereotypes that exist in people function as a bias that conditions their behavior. The presence of negative stereotypes towards older people has negative consequences both at an individual level and at a social level, since discriminatory practices occur towards this group. Therefore, the present project arises to respond a social problem detected by the National Institute of Social Services for Retirees and Pensioners (PAMI) of LaMatanza, Buenos Aires Argentina who had contacted a group of researchers of National Council of Scientific and Technical Research (CONICET) Argentina, to provide an answer to this problem in the region, were few studies were done about this subject. The aim of the present study is to analyze attitudes towards old age, and to design an intervention to reverse inequality and discrimination in the District of LaMatanza, Province of Buenos Aires.

**Method:**

Design: Prospective cohort study. Universe: Adults over 18 years old. Sample: 800 adults over 18 years (400 subjects between 18 and 59 years old and 400 over 60 years old) will be interviewed.

**Result:**

The project includes a research and intervention. It is expected to find different results that helps to work with the stereotypes in the three stages of the project 1) meetings with PAMI staff and members of the community to know their perspective, and select the data collection instruments 2) a prospective study will be carried out applying quantitative and qualitative instruments 3) Presentation of the final report and recommendations of possible interventions against ageism in the municipality.

**Conclusion:**

The World Health Organization has postulated that prejudices and negative stereotypes towards old age can interfere in vital aspects of being such as health, social participation, and security. It recognized ageism as a public health problem. It is expected in this research and intervention study, to find heterogeneity in the social attitudes of the different actors interviewed. The results will thus make it possible to detect the population sectors that require more work in relation to negative attitudes towards old age, as well as the population sectors that present more favorable attitudes and can be allies in the face of the necessary changes.

